# Mental health outcomes among Arab refugees, immigrants, and U.S. born Arab Americans in Southeast Michigan: a cross-sectional study

**DOI:** 10.1186/s12888-018-1948-8

**Published:** 2018-12-04

**Authors:** Sanjana Pampati, Zaineb Alattar, Evette Cordoba, Madiha Tariq, Carlos Mendes de Leon

**Affiliations:** 1 University of Michigan School of Public Health, 1415 Washington Heights, Ann Arbor, MI 48109 USA; 2 University of North Carolina Gillings School of Global Public Health, 135 Dauer Dr., Chapel Hill, NC 27599 USA; 3 Arab Community Center for Economic and Social Services (ACCESS), 2651 Saulino Ct., Dearborn, MI 48120 USA

**Keywords:** Refugee, Immigrant, Arab-American, Depression, Anxiety

## Abstract

**Background:**

Arab refugees and immigrants living in the United States may be exposed to political, economic, social, and environmental stressors that may affect their mental health. Yet, little is known regarding mental health outcomes among Arab Americans. The purpose of this study was to measure depression and anxiety levels among Arabs in Southeast Michigan and determine whether these levels differ by resident status: refugee, immigrant, or U.S. born.

**Methods:**

We conducted a cross-sectional study in a convenience sample of 275 adults who self-identify as Arab living in Southeast Michigan. Participants were recruited from a non-profit health and social services organization between August–November 2015. Data were collected via self-administered questionnaires, using standardized instruments to assess depression and anxiety symptoms.

**Results:**

All three resident groups exhibited high mean levels of depression and anxiety. Refugees reported higher levels of depression and anxiety than either immigrants or U.S. born Arab Americans. After adjustment for sociodemographics, differences between U.S. born Arab Americans and refugees were statistically significant for depression (b = 2.84; 95% CI: 0.21, 5.47), but not for anxiety. Refugees had significantly higher depression scores (b = 3.18, 95% CI: 1.52, 4.84) and anxiety scores (b = 1.31, 95% CI: 0.11, 2.50) than immigrants. Those reporting political violence and religious persecution as reasons for immigration had the highest levels of depression and anxiety.

**Conclusions:**

This convenience sample of Arab Americans reported high levels of depression and anxiety symptoms. Refugees appear to have poorer mental health outcomes than either immigrants or U.S.-born Arab Americans.

## Background

The overall purpose of this study is to provide an initial examination of mental health outcomes among Arab refugees, immigrants, and U.S. born Arab Americans in Southeast Michigan. Over the last few decades, the United States has become home to a growing population of refugees from Arab countries in the Middle East and North Africa (MENA) [1, 2]. The majority of those refugees in the past decade have fled war, political oppression, and persecution in Iraq and Syria [3, 4]. Given the many social, political, environmental, and economic stressors that refugees and immigrants may be exposed to before, during, and after their journey, this population may be at high risk of developing adverse mental health outcomes [5–11]. Pre-migration stressors can include war, political conflict, persecution based on religion, race, ethnicity, gender, sexual orientation, or other social group membership, and economic stress [5, 6, 12]. Post-migration stressors can include acculturative stress associated with resettlement, language barriers, social isolation, xenophobia, Islamophobia, discrimination, and difficulty accessing secure employment, housing, and transportation [5, 6, 12–14].

Many Arab refugees and immigrants enter and settle in Southeast Michigan, which is one of the largest recipient regions in the U.S. for this population [3, 15]. In the past decade (FY2008-FY2018), Michigan has received 35,009 refugees, including 20,362 Iraqis and 2256 Syrians; that is 14% of all Iraqi/Syrian refugees arriving to the U.S. during this period, and more than any other state after California [16]. Historically, most Arab refugees and immigrants in Michigan have settled around the Detroit Metro Area, which has been drawing immigrants going back to the late nineteenth century with the booming automotive industry [2]. Today, the city of Dearborn is home to the largest proportion of Arabs in the U.S., with Arab Americans representing 49.8% of the population [17]. There is very little systematic information on the mental health needs of the Arab-origin population in the U.S. The small number of studies thus far suggest that Arab Americans experience greater mental health challenges relative to majority non-Hispanic Whites. For example, data from the National Health Interview Survey indicate that foreign-born adults from the Middle East report significantly more serious psychological distress compared with foreign-born adults from Europe, or U.S.-born non-Hispanic Whites [18]. Data from a 2004 internet-based non-probability convenience sample of Arab Americans revealed much higher depression and anxiety levels in this population relative to normative data from non-Hispanic Whites and other ethnic groups, with about 50% reporting depression scores that met clinical case criteria, and about one-fourth reporting moderate to severe anxiety [19].

In addition, there may also be important mental health differences within the Arab American population itself, especially as a function of immigrant status (refugee, immigrant, or U.S. born). According to data from the 2003 Detroit Arab American Study, Arab immigrants reported poorer self-rated health than U.S. born Arab Americans [20]. Similarly, a study on older Arab Americans living in the Detroit Metro Area, age 56 and older, also found that immigrants report more frequent feelings of depression and lower life satisfaction than their U.S. born counterparts [21]. Further, among Arab Americans age 60 and older, higher levels of depression have also been found among refugees as compared to citizens, permanent residents, and visa holders [22]. Previous research in other immigrant populations suggests that refugees tend to have more mental health issues compared with immigrants. For example, Tamil refugees and asylum seekers reported more symptoms of anxiety, depression, and post-traumatic stress than Tamil immigrants [23]. Similarly, female refugees in Sweden had a significantly higher likelihood of purchasing psychotropic drugs, a proxy measure for mental health problems, compared to non-refugee immigrants [24]. Recent conflicts in the MENA region may put immigrants and refugees from that region at particular risk for adverse mental health outcomes, even relative to those with the same heritage but born in the U.S. However, the degree to which differences in country of birth (U.S. versus MENA) and refugee status affect mental health in Arab Americans is currently unknown.

In this study, we examine (1) the degree to which resident status (refugee, immigrant, U.S. born) is associated with depression and anxiety in Arab Americans, (2) whether duration living in the U.S. moderates the relationship between resident status and these mental health outcomes, and (3) the association between specific reasons for migration and each of these mental health outcomes among Arab immigrants and refugees.

## Methods

### Study background

This is a cross-sectional study of a convenience sample of adults who self-identify as Arab. Between August 2015 to November 2015, participants (*n* = 309) were recruited from the health and social services waiting room of the Arab Community Center for Economic and Social Services (ACCESS) in Dearborn, Michigan. ACCESS is the largest Arab American community nonprofit organization in the U.S. Founded in Dearborn, Michigan in 1971 with the purpose of helping Arab immigrants adjust to their new life in the U.S., it has since expanded and now provides a variety of health, education, employment, and social services to different communities in Southeast Michigan [25]. Although ACCESS’ services are open to a diverse population, clients tend to come from low-income backgrounds and are typically more recent immigrants and refugees. Eligibility for this study was restricted to those who were at least 18 years old and self-identified as being of Arab origin. Standardized English questionnaires covering basic demographic information, health conditions, mental health, and risk behaviors were translated into Modern Standard Arabic and back-translated into English independently by 2 bilingual study staff members. A consensus approach was used to resolve any differences. All participants completed either a self-administered English or Arabic-language paper questionnaire after providing written informed consent. IRB approval was received from the University of Michigan. Participants were compensated $10 for their time and effort.

#### Measures

Depression was measured using the Center for Epidemiologic Studies Depression Short Form (CES-D-SF), a shorter, 8 item version of the CES-D [26]. The CES-D-SF assesses the frequency of common depressive symptoms over the past 14 days, rated on a frequency scale as follows: not at all, several days, more than half the days, and nearly every day. The internal consistency of the CES-D-SF was found to be high (Cronbach coefficient alpha = 0.84). The CES-D has been successfully used to measure depression among Arab populations in the Middle East as well as Arab Americans [14, 27–29]. Summary scores of depressive symptomatology (referred to as depression scores) were calculated by summing the responses to the 8 items (range 0–24). We used mean imputation for participants with one missing item (*n* = 36). Depression scores for participants with ≥ 2 items missing were set to missing (*n* = 19). Anxiety was measured using 4 items from the Generalized Anxiety Disorder (GAD-4) scale [30]. The GAD-4 scale assesses the frequency of GAD symptoms over the past 14 days using the same 4-point scale as the CES-D-SF. The internal consistency of the GAD-4 instrument was high (Cronbach coefficient alpha = 0.90). Summary scores of anxiety symptomatology (referred to as anxiety scores) were calculated by summing the responses to the 4 items (range 0–12).

Resident status was ascertained based on 3 questions: “What country were you born in?”, “Do you have legal refugee status?”, and “If you were not born in the United States, in what year did you first enter the United States to live?” Participants who responded that they were born in the U.S. were categorized as U.S. born Arab Americans. Participants who responded that they had legal refugee status were categorized as refugees. Participants who listed another country besides the “United States” as their nation of birth or participants who reported a year of immigration to the U.S. and did not report legal refugee status were categorized as immigrants. We did not assess asylum status due to the very limited number of Arab-origin individuals granted asylum in the U.S. [3].

Duration living in the U.S. was modelled as a continuous variable, determined based on the reported year of immigration. For U.S. born Arab Americans, duration was set to their current age. Highest educational attainment was modelled as an ordinal variable and categorized as less than high school, high school/GED, college, or greater than college (graduate work). Marital status was categorized as married, divorced/separated, widowed, or never married, and modeled as married versus non-married in the regression analyses.

Reasons for immigrating to the U.S. were assessed by asking “If you immigrated to the United States, for what reason did you leave your home country?” Possible responses included political violence, religious persecution, seeking educational opportunity, seeking economic opportunity, and all of the above. These categories were not mutually exclusive and multiple responses could be selected. Participants were also permitted to write in a response. Write-in responses were either re-classified into one of the existing categories or were classified in the new response category “family reasons,” as it mostly included family reunification. U.S. born Arab Americans who responded to this question were set to missing.

#### Analysis

The analysis for this study was restricted to those with complete self-reported age, sex, and resident status information (*n* = 275). The sample consisted of refugees (*n* = 67), immigrants (*n* = 153), and U.S. born Arab Americans (*n* = 55). To test the association between resident status and depression and anxiety outcomes, we fitted a linear regression model for each outcome with U.S. born Arab Americans serving as reference category for resident status. Each model also included control variables for age, sex, duration living in the U.S., education, and marital status. In both models, interaction between resident status and duration living in the U.S. were tested to see if mental health outcomes associated with resident status varied as a function of time since arriving in the U.S. In order to test differences between refugees and immigrants, we repeated the aforementioned models with immigrants as reference group. All models were fitted in SAS 9.4. An exploratory analysis examining mean depression and anxiety levels by reason for immigration was also conducted.

## Results

### Participant characteristics

Of the 275 participants with complete sociodemographic information, the majority resided in Dearborn (*n* = 156), Dearborn Heights (*n* = 58), or Detroit (*n* = 18). Most of them identified as Muslim (*n* = 255), while a small minority identified as Christian (*n* = 11; data not shown). Of the 275 participants, several were missing information on relevant covariates, including marital status (*n* = 6), duration in the U.S. (*n* = 21), depression (*n* = 19), and anxiety (*n* = 4). Thus, the depression analysis included a sample size of 233 and the anxiety analysis included a sample size of 245. Sociodemographic characteristics of the total analytic sample (*n* = 275) and by resident status are shown in Table 1. Mean age for the total sample was 38.7 years (SD = 1.7 years; range = 18–85 years) and the majority was female (61.8%). Although the mean age of refugees (42.8 ± 3.4 years) and immigrants (41.2 ± 2.2 years) were similar, U.S. born Arab Americans were considerably younger (26.5 ± 3.2 years). Educational attainment varied based on resident status, with one third (34.3%) of refugees, 55% (46.4% + 8.5%) of immigrants, and three-quarters (61.8% + 14.6%) of U.S. born Arab Americans reporting a college degree or more. Most participants were married (58.0%), with U.S. born participants less likely to be married (24.5%) than either refugees (73.8%) or immigrants (62.9%). Refugees reported a shorter duration of being in the U.S. than immigrants; 39.7% of them had lived in the U.S. ≤ 5 years compared with 19.8% of immigrants. Mean depression scores were 8.85 (SD = 5.52) for the total sample, and 11.93 (SD = 5.35), 8.04 (SD = 5.46), and 7.39 (SD = 4.55) for refugees, immigrants, and U.S. born Arab Americans, respectively. Mean anxiety scores were 4.77 (SD = 3.77) for the total sample, and 5.88 (SD = 3.68), 4.41 (SD = 3.79), and 4.51 (SD = 3.65) for refugees, immigrants, and U.S. born Arab Americans, respectively.

**Table 1:** Characteristics of the Study Sample by Resident Status

	Total (*n* = 275), No. (%), or Mean (95% CI)	Refugee (n = 67), No. (%), or Mean (95% CI)	Immigrant (n = 153), No. (%), or Mean (95% CI)	U.S. Born (n = 55), No. (%), or Mean (95% CI)
Age, years	38.7 (36.9, 40.4)	42.8 (39.4, 46.3)	41.2 (39.0, 43.3)	26.5 (23.3, 29.8)
Female	170 (61.8%)	33 (49.2%)	97 (63%)	40 (72.7%)
Education				
< High school	45 (16.4%)	15 (22.4%)	27 (17.6%)	3 (5.4%)
High school/GED	81 (29.4%)	29 (43.3%)	42 (27.4%)	10 (18.2%)
College	128 (46.5%)	23 (34.3%)	71 (46.4%)	34 (61.8%)
> College	21 (7.6%)	0 (0.0%)	13 (8.5%)	8 (14.6%)
Marital Status				
Married	156 (58.0%)	48 (73.8%)	95 (62.9%)	13 (24.5%)
Divorced or separated	32 (11.9%)	5 (7.7%)	25 (16.6%)	2 (3.8%)
Widowed	11 (4.1%)	3 (4.6%)	7 (4.6%)	1 (1.9%)
Never married	70 (26.0%)	9 (13.8%)	24 (15.9%)	37 (69.8%)
Duration in U.S.				
≤ 5 years	52 (20.5%)	25 (39.7%)	27 (19.8%)	0 (0.0%)
6–14 years	53 (20.9%)	19 (30.2%)	34 (25.0%)	0 (0.0%)
≥ 15 years	149 (58.7%)	19 (30.2%)	75 (55.2%)	55 (100%)
Depression score	8.85 (5.52)	11.93 (5.35)	8.04 (5.46)	7.39 (4.55)
Anxiety score	4.77 (3.77)	5.88 (3.68)	4.41 (3.79)	4.51 (3.65)
Reasons for immigration^a^				
Political violence	95 (45.5%)	46 (69.7%)	49 (34.3%)	–
Religious persecution	39 (18.7%)	13 (19.7%)	26 (18.2%)	–
Educational opportunity	70 (33.5%)	10 (15.2%)	60 (42.0%)	–
Economic opportunity	91 (43.5%)	17 (25.8%)	74 (51.8%)	–
Familial reasons	24 (11.5%)	4 (6.1%)	20 (14.0%)	–

^a^Categories not mutually exclusive; participants could select more than one reason

#### Regression analyses

In regression analyses controlling for age, sex, marital status, education, and duration living in the U.S., refugee status was associated with significantly higher depression scores (b = 2.84; 95% CI: 0.21, 5.47) relative to U.S. born Arab Americans (Table 2, Model 1). Depression scores were not significantly different between immigrants and U.S. born Arab Americans. Using immigrants as the referent group in an additional model, we found that refugees had significantly higher depression scores than immigrants (b = 3.18, 95% CI: 1.52, 4.84; data not shown). Of the other covariates in the model, only educational attainment was significantly associated with depression scores, such that higher education was associated with lower depressive scores (b = − 0.99; 95% CI: -1.39, − 0.59). Interaction effects between duration living in the U.S. and refugee and immigrant status were tested but did not yield significant results (data not shown).

**Table 2:** Linear Regression Analyses Predicting Depression and Anxiety Scores

Variable	Model 1: Depression Score (*n* = 233) b (95% CI)	Model 2: Anxiety Score (*n* = 245) b (95% CI)
Resident status		
Refugee	2.84 (0.21, 5.47)	1.38 (−0.52, 3.27)
Immigrant	−0.34 (−2.43, 1.75)	0.07 (−1.44, 1.57)
U.S. Born (ref)	–	–
Age	0.05 (−0.02, 0.11)	0.01 (−0.04, 0.06)
Female	0.79 (−0.63, 2.22)	0.88 (−0.14, 1.89)
Married	−1.02 (−2.51, 0.47)	−0.47 (−1.54, 0.59)
Education	−0.99 (−1.39, −0.59)	−0.42 (−0.70, −0.14)
Duration in U.S.	−0.03 (−0.09, 0.04)	0.01 (−0.04, 0.05)

In regression analyses for anxiety scores controlling for the same set of covariates, neither refugee status (b = 1.38; 95% CI: -0.52, 3.27) nor immigrant status (b = 0.07; 95% CI: -1.44, 1.57) were associated with significantly different anxiety scores relative to U.S. born Arab Americans (see Table 2, Model 2). Using immigrants as the referent group in an additional model, we found that refugees had significantly higher anxiety scores than immigrants (b = 1.31, 95% CI: 0.11, 2.50; data not shown). Of the other covariates, only educational attainment was significantly associated with anxiety scores, such that higher education was associated with lower anxiety scores (b = − 0.42; 95% CI: -0.70, − 0.14). Interaction effects between duration living in the U.S. and refugee and immigrant status were tested in both models but did not yield significant results (data not shown).

#### Reasons for immigration

The exploratory analysis of mental health outcomes by reasons for immigration revealed that those reporting political violence and religious persecution had the highest levels of depression and anxiety (see Table 3). This was particularly the case if these were the only reasons they listed for immigration to the U.S. In contrast, those reporting educational opportunity or economic opportunity, especially as the sole reason for immigration, had the lowest levels of depression and anxiety. Immigrants and refugees reporting family reasons for coming to the U.S. had average levels of depression and anxiety in this sample.

**Table 3:** Depression and Anxiety Scores by Reason for Immigration

Reason for Immigration	Depression		Anxiety	
	N	Mean (SD)	N	Mean (SD)
Political violence only	49	10.90 (5.90)	52	5.21 (3.85)
Political violence and other	38	9.48 (6.16)	40	4.72 (3.53)
Religious persecution only	5	14.60 (1.95)	5	10.80 (1.30)
Religious persecution and other	32	9.40 (6.46)	34	4.47 (3.26)
Educational opportunity only	26	6.49 (5.13)	27	4.18 (4.00)
Educational opportunity and other	38	9.16 (6.48)	42	5.05 (3.87)
Economic opportunity only	46	8.92 (5.41)	50	3.96 (3.23)
Economic opportunity and other	36	9.02 (6.70)	40	4.92 (3.93)
Familial reasons only	22	9.50 (4.42)	24	4.96 (3.62)

## Discussion

Our findings suggest potentially important differences in mental health outcomes within the Arab American population. Overall, and not unexpectedly, Arab refugees appear to experience higher depression and anxiety symptom levels than U.S. born Arab Americans, even if these differences were statistically significant only for depression among refugees after adjustment for relevant correlates. Further, they also experienced higher depression and anxiety symptom levels than immigrants. Due to the stressors that Arab refugees may experience before and after resettlement, they may well be at particular risk of adverse mental health outcomes [5–11]. Other challenges that may contribute to higher depression among refugees compared to the U.S. born include difficulties in integrating into society and accessing opportunities in employment and other social domains [5, 6, 12, 13]. In addition, in an exploratory analysis, we found that those reporting political violence and religious persecution as reasons for immigration had the highest levels of depression and anxiety.

Our findings suggest that there is greater mental health suffering among Arab Americans not born in the U.S., especially those that came as refugees. The relatively lower depression scores found among U.S. born Arab Americans in this study are consistent with past findings of greater perceived well-being, including less frequent feelings of depression, among older U.S. born Arab Americans in the Detroit Metro Area, compared to immigrants [21]. According to their findings, access to human capital, such as language acquisition, may mediate the association between immigrant status and perceived well-being, with U.S. born Arab Americans’ relatively greater English proficiency facilitating more integration opportunities, and therefore contributing to greater well-being [21]. Although that study only compared immigrants and U.S. born Arab Americans, the potential mediating effect of human capital may still be relevant when comparing refugees and U.S. born Arab Americans. Other possible mediating factors may be differential exposure to stressors or sense of control between refugees and U.S. born Arab Americans.

Differences in mental health were also apparent between refugees and immigrants. This pattern is consistent with the small number of previous studies directly comparing refugees and immigrants [20, 23, 24], and speaks to the particular mental health challenges refugees face as they enter the U.S., regardless of country of origin. While adverse mental health outcomes did not appear to vary as a function of duration since initial entry into the U.S., this may be due to the relatively small number of refugees in our sample. This is consistent with another study which found that length of time in the U.S. was not significantly associated with well-being, or frequency of feeling depressed, among older Arab American immigrants in the Detroit Metro Area [21]. More systematic studies of the mental health risks in refugee populations are needed, including a focus on the severity and course of these mental health conditions after longer periods of settlement.

In an exploratory analysis, we found that refugees and immigrants reporting political violence or religious persecution as reasons for immigration had the highest levels of depression and anxiety, especially those who listed these as the only reason for coming to the U.S. These findings are consistent with other studies that have found an association between pre-migration exposure to political violence and subsequent negative mental health outcomes such as depression and post-traumatic stress disorder among both Arab and non-Arab populations [31–33]. We also found that Arab American immigrants reporting educational opportunity or economic opportunity as reasons for coming to the U.S. had relatively lower depression and anxiety levels. Taken together, this suggests that escaping political violence or religious persecution in one’s home country is more traumatizing and has more adverse mental health consequences than leaving one’s home country in search of educational or economic opportunity. Given the small sample size and the lack of a more systematic sampling method, these findings are suggestive at best, and larger and more detailed studies are urgently needed to examine these important aspects of Arab refugee and immigrant mental health. Nevertheless, these findings may underscore the need for ensuring adequate mental health services for immigrants and refugees, especially for those who have been exposed to political violence and religious persecution.

Overall, the findings further suggest that Arab Americans in Southeast Michigan experience high levels of depressive and anxiety symptoms. The average depression score in our sample was 8.85. While this symptom level cannot be compared directly to those reported in previous studies, it is worth noting that this depressive symptom level is substantially higher than those reported for other major U.S population groups based on a nationally representative sample using the identical measure, which reported depression scores of 3.96 for African Americans, 4.38 for Hispanics, and 3.27 for non-Hispanic Whites [34]. Similarly, anxiety levels in our sample of Arab Americans also appeared substantially higher than those reported previously. For example, the Detroit Neighborhood Health Study (DNHS), a population-based study of adult (≥ 18 years old) residents of Detroit (the same geographic area as the present study) using the identical anxiety measure reported mean anxiety scores of 1.27, 2.56, and 1.10 for African Americans, Hispanic and non-Hispanic White residents, respectively (unpublished data). These levels compare to a mean anxiety score of 4.77 that we found in our sample of Arab Americans.

Overall, our findings are suggestive of considerable mental health needs in the Arab American population in Southeast Michigan, which could be due to a number of reasons. Since 9/11, there have been an increasing number of anti-Arab incidents of discrimination, such as ethnic-based harassment and hate crimes as well as discrimination in employment, housing, and other services [19]. Additionally, the majority of our sample is Muslim, putting them at added risk for Islamophobia-motivated forms of discrimination. The fear and social isolation that may result from this discrimination may well have contributed to the high levels of depression and anxiety across all Arab Americans in our sample. While these findings are limited to Arab Americans who reside in Southeast Michigan, this area is one of the largest population centers of Arab Americans in the country, including those who settled several generations ago as well as more recent immigrants and refugees. They are also consistent with previous research that have found higher levels of depression among Arab Americans relative to other ethnic groups in the Detroit Metro Area [31]. Our findings also corroborate those of Amer et al. (2011), who found higher levels of depression and anxiety in a large geographically and socio-demographically diverse sample of Arab Americans compared to other minority groups [19]. Taken together, these findings emphasize the importance of public health research to recognize the growing Arab population in the U.S. as a distinct entity and the unique mental health challenges they may face.

There are several limitations of this study. The measures to capture depression and anxiety have not been specifically validated for use in Arab Americans. The CESD-SF and GAD-4 focus primarily on mood, such as feelings of sadness or nervousness. However, it is possible that Arab Americans experience symptoms of depression and anxiety in slightly different ways; for example, Arab Americans may focus more on physical attributes such as chest tightness, body aches, and fatigue to convey experiences of depression and anxiety, as has been noted in other Asian populations as well [35–37]. Further, due to the stigma associated with mental illness, both generally and particularly in Arab communities, participants may have been reluctant to respond honestly to questions about depression and anxiety [38–40]. While the high levels of depression and anxiety symptoms we observed in our sample make under-estimation of these symptoms less of a concern, more research is needed with measures that are more sensitive to the cultural nuances with which mental disorders are expressed in Arab-origin populations, including Arab Americans. In addition, our measure of resident status did not assess asylum status. However, a comparatively low number of Arab-origin individuals are granted asylum status in the U.S. [4]. We were also unable to validate participants’ self-reported refugee status. It may be possible that individuals did not want to share their refugee status due to stigma or were not certain about their own status. Further, many individuals likely meet the U.S. criteria for determining refugee status but never formally applied for it. It is also important to note that data for this study came from a convenience sample of Arab Americans recruited from ACCESS, which provides health and social services to predominantly low-income clients. As a result, our findings are not necessarily generalizable to the entire Arab American population in Southeast Michigan. However, ACCESS’ client-base does include people with a range of sociodemographic backgrounds, which is reflected in our sample which similarly includes participants with a broad spectrum of background characteristics such as age, marital status, and educational attainment. In addition, the sample consisted of Arab Americans who differed in resident status, including refugees, immigrants, and U.S. born residents. Still, it is possible that this sample has greater health needs than the Arab American population more generally, and this may have contributed to the high levels of depression and anxiety symptoms found in this study. Additionally, nearly all of the sample identified as Muslim (93%), likely due to the large population of Muslim Arabs in Dearborn where ACCESS is located. However, this is not representative of the larger Arab American community in Southeast Michigan which also includes a substantial Christian population, further limiting the generalizability of our findings. It is important to note that recruiting a random sample is difficult, as the U.S. Census does not include an Arab or Middle East and North Africa (MENA) identifier. More systematic studies in this population are urgently needed, and would be greatly facilitated by the re-introduction of Arab-origin or MENA ethnicity identifiers on the U.S. Census. Nonetheless, our study serves as an initial investigation of the mental health needs in the Arab American population.

In addition, although the findings are based on cross-sectional data, reporting errors in resident status or other key variables due to mental health seem an unlikely source of bias. Although the survey was administered anonymously, participants may have been reluctant to share potentially stigmatizing (e.g., depressive thoughts) or criminalizing (e.g., unlawful resident status) information due to fear of government surveillance or disclosure of information to their community. Also, no information was available on pre-immigration experiences to specify or validate the circumstances that led to decisions to immigrate, which prevented a more in-depth analysis of the role of pre-immigration exposures in post-settlement mental health. Future studies should conduct a more in-depth evaluation of mental health needs of Arab Americans. Despite these limitations, this is one of a very limited number of studies to assess the mental health of the growing Arab refugee and immigrant population in the U.S.

## Conclusions

In sum, this study provides important new information on mental health outcomes of Arab-origin adults in the U.S. The findings suggest that our sample of Arab Americans report very high levels of depression and anxiety symptoms. In particular, Arab-origin refugees and immigrants who have resettled in the U.S. to escape political violence or religious persecution experience higher levels of depression and anxiety compared to those immigrating for other reasons. While such pre-migration stressors are not the focus of this paper, mental health outcomes among immigrants and refugees may also be affected by stressors that occur after resettlement, such as discrimination, social isolation, and difficulty obtaining employment. In order to make mental health intervention and prevention efforts more effective in this population, future research should aim to identify the unique stressors that contribute to these adverse mental health outcomes in Arab Americans. With the growing population of Arab Americans more generally, including Arab-origin immigrants and refugees in the U.S., more systematic efforts are required to examine and address the scope and determinants of their mental health needs.

## Abbreviation

MENA: Middle East and North Africa
